# MRI brain volume loss, lesion burden, and clinical outcome in secondary progressive multiple sclerosis

**DOI:** 10.1177/13524585211031801

**Published:** 2021-07-26

**Authors:** Marcus W Koch, Jop Mostert, Pavle Repovic, James D Bowen, Eva Strijbis, Bernard Uitdehaag, Gary Cutter

**Affiliations:** Department of Clinical Neurosciences, University of Calgary, Calgary, AB, Canada/Department of Community Health Sciences, University of Calgary, Calgary, AB, Canada; Department of Neurology, Rijnstate Hospital, Arnhem, The Netherlands; Multiple Sclerosis Center, Swedish Neuroscience Institute, Seattle, WA, USA; Multiple Sclerosis Center, Swedish Neuroscience Institute, Seattle, WA, USA; Department of Neurology, MS Center Amsterdam, Amsterdam University Medical Centers, Amsterdam, The Netherlands; Department of Neurology, MS Center Amsterdam, Amsterdam University Medical Centers, Amsterdam, The Netherlands; Department of Biostatistics, The University of Alabama at Birmingham, Birmingham, AL, USA

**Keywords:** Multiple sclerosis, progressive multiple sclerosis, magnetic resonance imaging (MRI), brain atrophy, outcome measures, clinical trial

## Abstract

**Background:**

Magnetic resonance imaging (MRI) of brain volume measures are widely used outcomes in secondary progressive multiple sclerosis (SPMS), but it is unclear whether they are associated with physical and cognitive disability.

**Objective:**

To investigate the association between MRI outcomes and physical and cognitive disability worsening in people with SPMS.

**Methods:**

We used data from ASCEND, a large randomized controlled trial (*n* = 889). We investigated the association of change in whole brain and gray matter volume, contrast enhancing lesions, and T2 lesions with significant worsening on the Expanded Disability Status Scale (EDSS), Timed 25-Foot Walk (T25FW), Nine-Hole Peg Test (NHPT), and Symbol Digit Modalities Test (SDMT) with logistic regression models.

**Results:**

We found no association between MRI measures and EDSS or SDMT worsening. T25FW worsening at 48 and 96 weeks, and NHPT worsening at 96 weeks were associated with cumulative new or newly enlarging T2 lesions at 96 weeks. NHPT worsening at 48 and 96 weeks was associated with normalized brain volume loss at 48 weeks, but not with other MRI outcomes.

**Conclusion:**

The association of standard MRI outcomes and disability was noticeably weak and inconsistent over 2 years of follow-up. These MRI outcomes may not be useful surrogates of disability measures in SPMS.

## Introduction

Focal inflammatory disease activity in multiple sclerosis (MS) can be seen on serial magnetic resonance imaging (MRI) scans as increasing T2 lesion number and volume,^1^ and the steady loss of neurons and glial cells presents as progressive loss of brain volume. Brain volume loss occurs in all forms of MS, even in radiologically isolated syndrome,^2^ before the onset of MS-related symptoms, but it is thought to be especially relevant in secondary progressive MS (SPMS), where diffuse neurodegeneration plays a more prominent pathophysiological role.^3^

MRI brain volume measures are widely used as outcome measures in clinical trials, including as the primary outcome measure in several phase 2 trials,^4–6^ likely with the underlying rationale that such measures could serve as useful biomarkers of disability worsening. Despite the biological plausibility of this approach, it should be kept in mind that brain volume loss is a slow process, developing over years to decades. It is unclear whether the relatively small brain volume changes measured over the 2 years of a typical clinical trial in SPMS are associated with significant physical and cognitive disability.

In this study, we used patient-level clinical and MRI outcome data from ASCEND, a large phase 3 study of natalizumab treatment in SPMS, to investigate the relation of MRI changes with significant worsening of physical and cognitive disability.

## Materials and methods

### ASCEND dataset

The ASCEND dataset is described in detail in the original publication of the trial.^7^ Briefly, ASCEND was a randomized, double blind, placebo-controlled, two-arm trial of natalizumab treatment in SPMS. The inclusion criteria were of age 18–58 years inclusive, SPMS for 2 or more years, disability progression over the previous year, a screening EDSS score of 3.0–6.5 inclusive, and a Multiple Sclerosis Severity Score^8^ of 4 or more. It excluded patients with a clinical relapse in the 3 months before inclusion. In ASCEND, SPMS was defined as relapsing-remitting disease followed by progression of disability independent of or not explained by MS relapses for at least 2 years.

### MRI outcomes

Gadolinium enhanced cranial MRI scans were performed at the screening visit of the trial, and then at 24, 48, 72, and 96 weeks of follow-up. Normalized brain volume (NBV), normalized cortical gray matter volume (NCGMV), and normalized whole gray matter volume (NWGMV) were determined using SIENAX, a segmentation-based cross-sectional method.^9^ The Jacobian integration technique was used to generate percent brain volume change, percent whole GM volume change, and percent cortical GM volume change on 3-mm thick slices. T2 lesion volume, and the number and volume of contrast enhancing lesions were assessed for all scans, and the number of new or newly enlarging T2 lesions for all scans after screening. We determined the cumulative number of contrast enhancing lesions (cCEL) and the cumulative number of new or newly enlarging T2 lesions (cNT2) at 24, 48, 72, and 96 weeks.

### Clinical outcomes

EDSS, T25FW, and NHPT were measured at the screening and baseline visit and then every 12 weeks. SDMT was measured at baseline and then every 4 weeks. For this study, we used significant worsening of disability with 3-month confirmation (3 month confirmed disability progression, 3M CDP) measured at the main study visits every 12 weeks. We determined the percentage of individuals with significant worsening of disability by comparing the screening and the follow-up measurement at each timepoint for the EDSS, T25FW (average of two trials) and NHPT (average of four trials, two for each hand), and between the baseline and the follow-up measurement at each timepoint for the SDMT. Individuals missing a measurement at screening (or baseline for SDMT), the follow-up time point of interest, or the corresponding 3-month confirmation assessment were excluded from the analysis. We defined significant worsening on the EDSS as an increase of one whole point on the EDSS if the screening EDSS was 5.5 or lower, and of one-half point if the screening EDSS was 6.0 or 6.5 (this definition was used in the original trial). For the T25FW and NHPT, we defined significant worsening as a 20% or greater increase from screening. We used a four-point decrease in the SDMT score as significant worsening, since this margin of worsening is associated with loss of employment in people with MS and generally seen as clinically significant.^10^

### Association of MRI outcomes with significant disability worsening

In a first step, we explored significant differences in the change in MRI outcomes at 48 and 96 weeks between participants with and without significant disability worsening at 48 and 96 weeks using Student’s *t*-test.

We then used logistic regression models to assess the association of 3M CDP on the clinical outcome measures (dependent variable) and MRI measures of interest (independent predictor variable). Additional independent predictor variables included in the models were: age, sex, treatment arm, and the MRI outcome of interest at screening. We categorized the change in volume measures NBV, NCGMV, and NWGMV into five categories: (1) volume increase or no change, (2) up to 0.5% volume loss, (3) between 0.5% and 1% volume loss, (4) between 1% and 1.5% volume loss, and (5) more than 1.5% volume loss. We categorized cNT2 into four categories: (1) None, (2) 1 to 5, (3) 6 to 10, and (4) more than 10. To achieve the greatest sensitivity for discovering associations, we chose not to correct significance levels for multiple comparisons. We used the R statistical software package for Windows version 4.0.2^11^ for all statistical analyses. Statistical significance was taken to be at the two-tailed 0.05 level.

### Data availability

The data used in this study are available upon request from Biogen. Individual participant data collected during the trial will be shared after anonymization and on approval of a research proposal and data sharing agreement. Research proposals can be submitted online (www.biogenclinicaldatarequest.com).

## Results

### ASCEND dataset

The ASCEND dataset contained data on 889 patients. Table 1 shows their baseline characteristics.

**Table 1.** Screening clinical and imaging characteristics of the ASCEND dataset.

Number of participants	889
Sex (f/m, %)	550 (61.9%)/339 (38.1%)
Age (median, IQR)	48, 42–53
EDSS at screening (median, IQR)	6.0, 5.0–6.5
T25FW at screening (median, IQR)	11.2, 8.0–17.0
NHPT at screening (median, IQR)	30.3, 25.5–38.8
SDMT at baseline (median, IQR)	39, 30–49
Patients with enhancing lesions at screening (*n*, %)	212, 23.9%^a^
NBV (cm^3^) (mean, SD)	1423.9, 83.3
NCGMV (cm^3^) (mean, SD)	513.9, 53.0
NWGMV (cm^3^) (mean, SD)	684.9, 63.8
T2 lesion volume (cm^3^) (mean, SD)	16.9, 17.5

IQR: interquartile range; EDSS: Expanded Disability Status Scale; T25FW: Timed 25-Foot Walk; NHPT: Nine-Hole Peg Test; SDMT: Symbol Digit Modalities Test; NBV: normalized brain volume; SD: standard deviation; NCGMV: normalized cortical gray matter volume; NWGMV: normalized whole gray matter volume.

a *n* = 888.

### MRI outcomes

**Figure 1.**
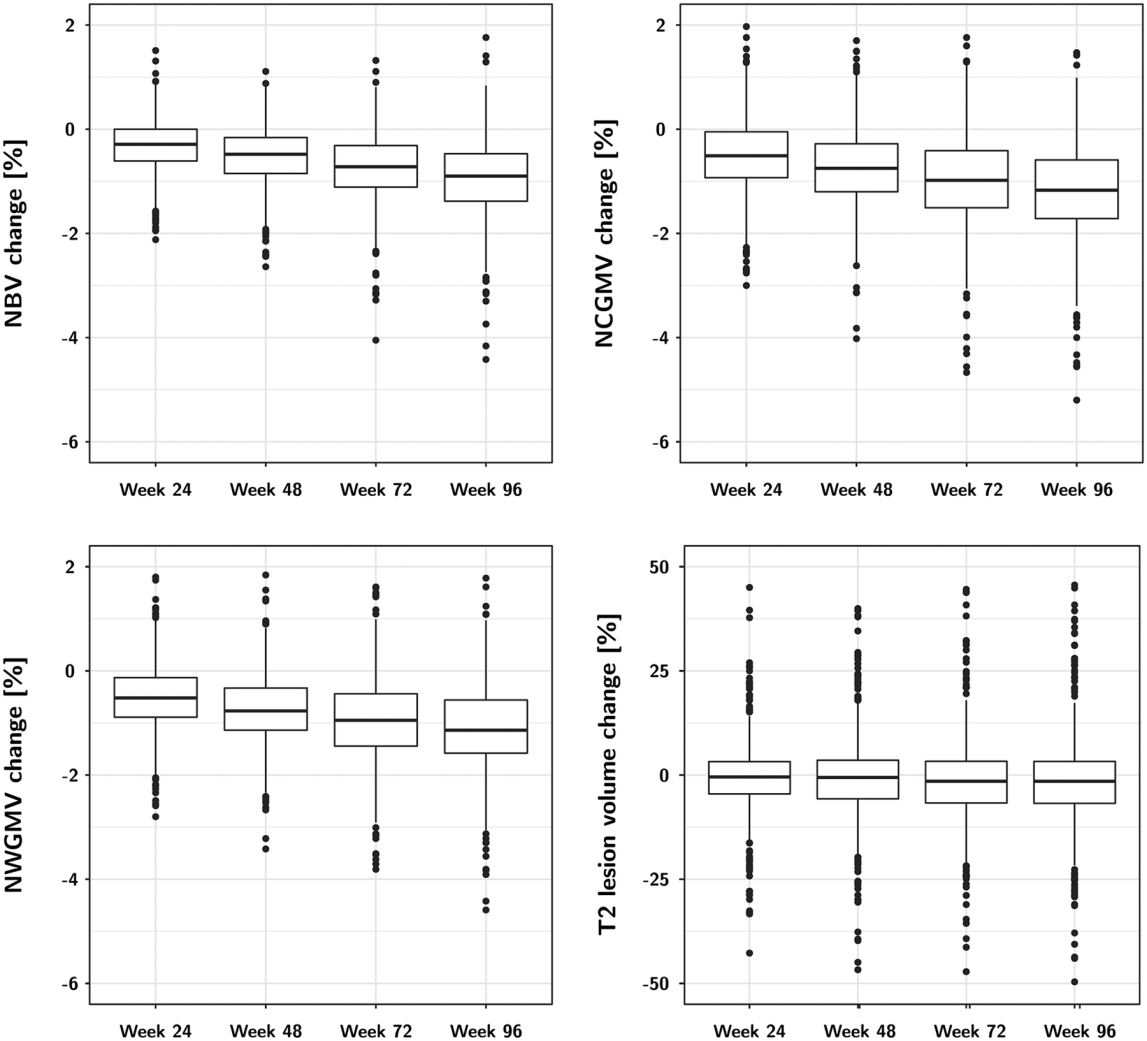
MRI volume changes between screening and follow-up MRI scans.

Change in the investigated MRI outcomes is shown in Table 2 and Figure 1. NBV, NCGMV, and NWGMV steadily decreased throughout follow-up reaching a mean volume loss of around 1% on all of these volume measures at 96 weeks, whereas T2 lesion volume changed little during follow-up (Table 2, Figure 1). The cCEL and the cNT2 steadily increased throughout follow-up (Table 2). All measures also showed slight increases in the variability of the changes.

**Table 2.** Changes in clinical and MRI outcomes over 2 years of follow-up.

Outcome	24 weeks	48 weeks	72 weeks	96 weeks
EDSS 3M CDP:				
Percentage	6.8	11.7	14.1	17.7
Participants with EDSS 3M CDP	52	81	93	111
Number of observations	766	690	658	627
T25FW 3M CDP				
Percentage	17.9	25.6	25.7	28.6
Participants with EDSS 3M CDP	134	169	158	165
Number of observations	747	661	615	577
NHPT 3M CDP				
Percentage	4.1	5.7	6.4	8.2
Participants with EDSS 3M CDP	30	37	40	49
Number of observations	728	650	621	597
SDMT 3M CDP				
Percentage	3.4	2.7	3.3	3.2
Participants with EDSS 3M CDP	25	18	21	19
Number of observations	728	658	630	597
NBV change (%, SD)	−0.32 (0.5)	−0.53 (0.57)	−0.75 (0.68)	−0.95 (0.76)
Number of participants by NBV change (*n*, %):				
⩾0%	194 (25.6)	110 (16.5)	70 (11.4)	45 (8.2)
<0 to −0.5%	316 (41.6)	233 (34.9)	154 (25.4)	103 (18.8)
<−0.5 to −1%	181 (23.8)	215 (32.2)	191 (31.5)	163 (29.7)
<−1 to −1.5%	52 (6.9)	74 (11.1)	126 (20.8)	132 (24.0)
<−1.5%	16 (2.1)	36 (5.4)	66 (10.9)	106 (19.3)
NCGMV change (%, SD)	–0.49 (0.72)	–0.74 (0.77)	–0.99 (0.91)	–1.18 (0.96)
NWGMV change (%, SD)	–0.51 (0.65)	–0.73 (0.69)	–0.96 (0.81)	–1.13 (0.86)
T2 lesion volume change (%, SD)	–0.39 (9.07)	–0.13 (13.11)	–0.71 (15.21)	–0.55 (15.01)
cCEL:				
Mean, SD	1.34 (5.25)	1.63 (6.65)	1.93 (8.48)	2.21 (10.3)
Median, IQR	0 (0–1)	0 (0–1)	0 (0–1)	0 (0–1)
cNT2:				
Mean, SD	1.54 (4.32)	2.4 (6.71)	3.18 (8.77)	3.7 (10.04)
Median, IQR	0 (0–1)	0 (0–2)	0 (0–2)	0 (0–3)
Number of participants by cNT2 (*n*, %):				
None	546 (67.7)	462 (63.1)	417 (62.0)	371 (59.2)
1–5	202 (25.0)	189 (25.8)	156 (23.2)	151 (24.1)
6–10	27 (3.3)	36 (4.9)	43 (6.4)	45 (7.2)
More than 10	32 (4.0)	45 (6.1)	57 (8.5)	60 (9.6)

EDSS: Expanded Disability Status Scale; 3M: 3 months; CDP: confirmed disability progression; T25FW: Timed 25-Foot Walk; NHPT: Nine-Hole Peg Test; SDMT: Symbol Digit Modalities Test; NBV: normalized brain volume; SD: standard deviation; NCGMV: normalized cortical gray matter volume; NWGMV: normalized whole gray matter volume; cCEL: cumulative number of contrast enhancing lesions; IQR: interquartile range; cNT2: cumulative number of new or newly enlarging T2 lesions.

### Clinical outcomes

**Figure 2.**
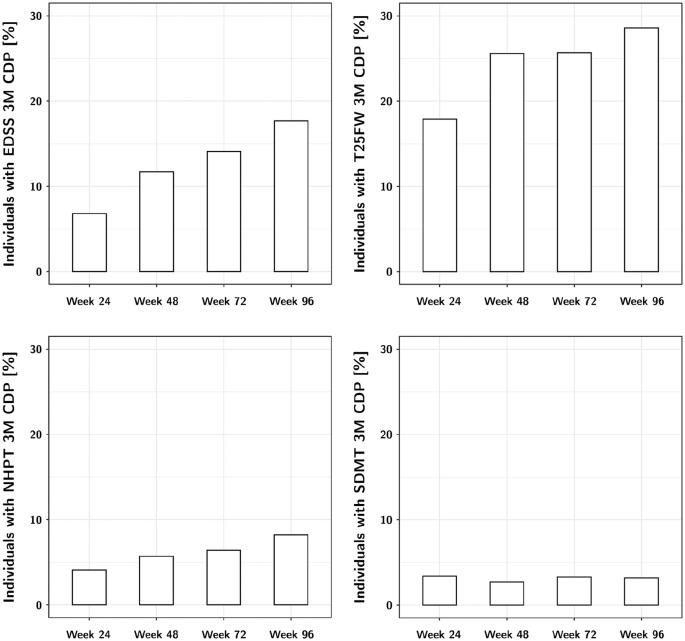
Proportion of individuals with 3-month confirmed disability worsening on the investigated measures throughout the trial.

Change in the investigated clinical outcome measures over the 2 years of follow-up is shown in Table 2 and Figure 2. The number of participants with significant worsening on the EDSS, T25FW and NHPT steadily increased throughout the course of the trial, while there was little change in SDMT. The T25FW had the most worsening events, followed by the EDSS and NHPT.

### Association of MRI outcomes with significant disability worsening

The unadjusted comparisons of change in MRI outcomes between patients with and without significant disability worsening are shown in Table 3. The NHPT was most consistently associated with MRI outcomes, with a greater amount of NBV, NCGMV, and NWGMV loss in patients with NHPT worsening at 48 weeks, and a greater amount of NBV loss, T2 lesion volume increase, and cNT2 at 96 weeks (Table 3).

**Table 3.** Differences in MRI outcomes between patients with and without significant disability worsening at 48 and at 96 weeks.

		NBV change (%)		NCGMV change (%)		NWGMV change (%)		T2 lesion volume change (%)		cCEL		cNT2	
		Mean (*SD*)	*p*	Mean (*SD*)	*p*	Mean (*SD*)	*p*	Mean (*SD*)	*p*	Mean (*SD*)	*p*	Mean (*SD*)	*p*
		48 weeks											
EDSS 3M CDP	Yes	−0.54 (0.62)	0.82	−0.80 (0.93)	0.51	−0.79 (0.82)	0.27	−0.72 (16.35)	0.85	2.67 (12.59)	0.41	3.58 (11.15)	0.24
	No	−0.53 (0.56)		−0.72 (0.75)		−0.72 (0.67)		−0.38 (12.36)		1.50 (5.65)		2.09 (5.41)	
T25FW 3M CDP	Yes	−0.58 (0.53)	0.12	−0.79 (0.76)	0.19	−0.78 (0.69)	0.25	−0.74 (11.83)	0.88	1.95 (6.53)	0.27	2.79 (5.94)	0.10
	No	−0.50 (0.58)		−0.70 (0.77)		−0.70 (0.68)		−0.57 (13.26)		1.33 (5.18)		1.92 (5.46)	
NHPT 3M CDP	yes	−0.86 (0.75)	0.02	−1.15 (0.99)	0.03	−1.08 (0.89)	0.03	3.58 (16.9)	0.19	1.77 (3.84)	0.71	2.90 (5.84)	0.46
	No	−0.51 (0.56)		−0.71 (0.75)		−0.70 (0.66)		−0.53 (12.93)		1.49 (5.65)		2.08 (5.43)	
SDMT 3M CDP	Yes	−0.68 (0.61)	0.47	−0.65 (0.47)	0.45	−0.70 (0.37)	0.76	−5.39 (7.11)	0.007	1.83 (4.46)	0.90	1.61 (2.09)	0.18
	No	−0.53 (0.58)		−0.74 (0.77)		−0.73 (0.69)		−0.16 (13.14)		1.69 (7.07)		2.37 (6.57)	
		96 weeks											
EDSS 3M CDP	Yes	−1.12 (0.81)	0.02	−1.35 (1.04)	0.06	−1.28 (0.92)	0.05	0.99 (16.55)	0.23	3.25 (13.06)	0.36	6.02 (16.69)	0.08
	No	−0.90 (0.74)		−1.13 (0.95)		−1.08 (0.85)		−1.06 (14.42)		2.02 (9.83)		3.17 (7.83)	
T25FW 3M CDP	Yes	−0.98 (0.70)	0.14	−1.22 (0.96)	0.31	−1.16 (0.86)	0.36	−0.76 (15.56)	0.52	3.08 (15.08)	0.21	4.45 (10.87)	0.08
	No	−0.88 (0.73)		−1.12 (0.97)		−1.08 (0.85)		−1.62 (12.24)		1.55 (5.68)		2.81 (7.63)	
NHPT 3M CDP	Yes	−1.47 (1.12)	0.002	−1.40 (1.13)	0.16	−1.31 (1.06)	0.21	4.68 (16.76)	0.02	3.26 (7.24)	0.27	7.81 (14.66)	0.03
	No	−0.88 (0.69)		−1.14 (0.97)		−1.09 (0.85)		−1.26 (14.73)		1.98 (9.57)		3.05 (7.99)	
SDMT 3M CDP	Yes	−0.96 (0.75)	0.89	−1.13 (0.79)	0.82	−0.99 (0.76)	0.49	−0.16 (10.63)	0.82	1.74 (3.03)	0.64	3.42 (4.50)	0.87
	No	−0.93 (0.76)		−1.18 (0.98)		−1.12 (0.87)		−0.73 (15.10)		2.12 (9.58)		3.61 (9.07)	

MRI: magnetic resonance imaging; NBV: normalized brain volume; NCGMV: normalized cortical gray matter volume; NWGMV: normalized whole gray matter volume; cCEL: cumulative number of contrast enhancing lesions; cNT2: cumulative number of new or newly enlarging T2 lesions; SD: standard deviation; EDSS: Expanded Disability Status Scale; 3M: 3 months; CDP: confirmed disability progression; T25FW: Timed 25-Foot Walk; NHPT: Nine-Hole Peg Test; SDMT: Symbol Digit Modalities Test.

After adjustment for other co-variables in the logistic regression models, we found few significant associations between clinical outcomes and MRI measures. Table 4 shows a summary of the results of all logistic regression models. Table 5 shows a summary of three selected logistic regression models with significant associations between MRI outcomes and significant disability worsening.

**Table 4.** Results of the logistic regression models investigating the association of MRI outcomes and clinical outcomes.

Predictor variable	Outcome							
	EDSS 3M CDP		T25FW 3M CDP		NHPT 3M CDP		SDMT 3M CDP	
	48 weeks	96 weeks	48 weeks	96 weeks	48 weeks	96 weeks	48 weeks	96 weeks
NBV change at 48 weeks (%)	No	No	No	No	**Yes**	**Yes**	No	No
NBV change at 96 weeks (%)	No	No	No	No	No	No	No	No
NCGMV change at 48 weeks (%)	No	No	No	No	No	No	No	No
NCGMV change at 96 weeks (%)	No	No	No	No	No	No	No	No
NWGMV change at 48 weeks (%)	No	No	No	No	No	No	No	No
NWGMV change at 96 weeks (%)	No	No	No	No	No	No	No	No
T2 lesion volume change at 48 weeks (%)	No	No	No	No	No	No	No	No
T2 lesion volume change at 96 weeks (%)	No	No	No	No	No	No	No	No
cCEL at 48 weeks	No	No	No	No	No	No	No	No
cCEL at 96 weeks	No	No	No	No	No	No	No	No
cNT2 at 48 weeks	No	No	No	No	No	No	No	No
cNT2 at 96 weeks	No	No	**Yes**	**Yes**	No	**Yes**	No	No

MRI: magnetic resonance imaging; EDSS: Expanded Disability Status Scale; 3M: 3 months; CDP: confirmed disability progression; T25FW: Timed 25-Foot Walk; NHPT: Nine-Hole Peg Test; SDMT: Symbol Digit Modalities Test; NBV: normalized brain volume; NCGMV: normalized cortical gray matter volume; NWGMV: normalized whole gray matter volume; cCEL: cumulative number of contrast enhancing lesions; cNT2: cumulative number of new or newly enlarging T2 lesions. The table answers the question of whether there is a significant association between the predictor variable (left column) of interest and the clinical outcomes EDSS, T25FW, NHPT, or SDMT. The models include the clinical outcome measure as the outcome variable (dependent variable) and the MRI measure of interest at 48 or 96 weeks as well as age, sex, treatment arm, and the MRI outcome of interest at screening as predictor (independent) variables.

**Table 5.** Detailed results from three selected logistic regression models.

Predictor variables	Odds ratio	95% confidence interval	*p*
NBV at 48 weeks and NHPT 3M CDP at 96 weeks			
NHPT at screening (s)^a^	1.01	0.99–1.02	0.38
Male sex (reference: female)	0.78	0.35–1.63	0.52
Age (years)^a^	0.97	0.93–1.01	0.19
Trial arm: natalizumab (reference: placebo)	1.03	0.51–2.12	0.93
NBV at screening (mL)^a^	1.00	0.99–1.01	0.62
NBV change to 48 weeks:			
⩾0% (reference)	1.00	(Reference)	–
<0 to −0.5%	0.90	0.23–4.34	0.88
<−0.5 to −1%	1.84	0.55–8.31	0.36
<−1 to −1.5%	4.39	1.23–20.64	0.03
<−1.5%	4.69	1.02–25.23	0.05
cNT2 at 96 weeks and T25FW 3M CDP at 96 weeks			
T25FW at screening (s)^a^	1.02	0.99–1.05	0.08
Male sex (reference: female)	0.76	0.50–1.13	0.19
Age (years)^a^	0.99	0.96–1.01	0.32
Trial arm: natalizumab (reference: placebo)	1.24	0.80–1.94	0.34
cNT2 at 96 weeks:			
None (reference)	1.00	(Reference)	–
1–5	1.34	0.82–2.18	0.24
6–10	1.57	0.71–3.37	0.25
More than 10	2.25	1.06–4.75	0.03
cNT2 at 96 weeks and NHPT 3M CDP at 96 weeks			
NHPT at screening (s)^a^	1.01	1.00–1.02	0.03
Male sex (reference: female)	1.48	0.79–2.72	0.21
Age (years)^a^	0.98	0.94–1.02	0.22
Trial arm: natalizumab (reference: placebo)	0.75	0.35–1.60	0.46
cNT2 at 96 weeks:			
None (reference)	1.00	(Reference)	–
1–5	1.25	0.54–2.80	0.59
6–10	1.45	0.37–4.60	0.56
More than 10	3.04	1.11–8.24	0.03

NBV: normalized brain volume; NHPT: Nine-Hole Peg Test; 3M: 3 months; CDP: confirmed disability progression; cNT2: cumulative number of new or newly enlarging T2 lesions; T25FW: Timed 25-Foot Walk.

a Per unit increase.

EDSS and SDMT worsening were not associated with any of the investigated MRI outcomes. Significant disability worsening on the T25FW at 48 and 96 weeks and on the NHPT at 96 weeks was associated with the cNT2 96 weeks, with an increasing number of T2 lesions associated with a greater risk of disability worsening (Table 5). The regression model for T25FW worsening at 48 weeks showed similar results (data not shown). Remarkably, these associations exist for the cNT2 at 96 weeks, but not for the cNT2 at 48 weeks.

Significant disability worsening on the NHPT at 48 and 96 weeks was also associated with NBV loss at 48 weeks, with greater volume loss associated with a greater risk of disability worsening (Table 5). The regression model for NHPT worsening at 48 weeks showed similar results (data not shown). Notably, these associations exist for NBV loss at 48 weeks, but not for NBV loss at 96 weeks.

### Additional analyses

We explored the possible influence of “selective drop-outs,” in the sense that participants with the largest change in MRI outcomes (T2 lesion volume, cCES, cNT2, NBV, NCGMV, or NWGMV) may have been more likely to drop out of the trial, which may have impacted the results. We used Student’s *t*-test to compare change in MRI outcomes at 48 weeks in patients with and without a subsequent measurement at 96 weeks. There were no statistically significant differences in MRI outcomes between any of these groups (data not shown).

## Discussion

Most studies on the association of MRI outcomes and disability in MS are cross-sectional or of short duration and investigate correlations and associations of brain volume, lesion volume, and lesion number with disability measures. Such studies have generally found statistically significant associations between MRI outcomes and disability measures.^12–15^ There are comparatively few longitudinal studies on brain volume loss and disability worsening. Several smaller longitudinal studies with follow-up durations ranging from 10 to 20 years^16–18^ showed that gray matter volume loss is more pronounced than white matter volume loss in the long term, and more closely associated with disability worsening in all forms of MS.

In contrast to these cross-sectional studies, we found a disconnect between the change in MRI measures and clinical outcomes over 2 years, even though we made an effort to be as sensitive as possible by using cumulative lesion numbers and by not adjusting for multiple comparisons. For the most established and widely used physical outcome measure EDSS, currently the standard primary outcome measures in phase 3 trials in all forms of MS, we found no significant associations with any of the investigated MRI measures, neither of brain volume measures nor of measures of lesion burden. The few associations between MRI measures and clinical outcomes we found were with the newer and possibly more sensitive outcomes T25FW and NHPT, but it is unclear if these associations are clinically meaningful.

Cognitive dysfunction is a common and impactful contributor to disability in MS. Global and regional brain volume loss are believed to be especially relevant and strongly related to cognitive function, as shown in smaller studies.^12,19^ Similar to physical outcome measures, there is a lack of large longitudinal studies to assess the association of MRI outcomes and cognitive function in SPMS. Similarly to our findings on physical disability measures, we found little change over 2 years for the SDMT, an established and patient-friendly cognitive outcome in MS that is recommended for standard clinical practice,^20^ and no association of significant change on the SDMT with any of the MRI measures. This is somewhat counterintuitive given the prominence of brain volume loss as an imaging characteristic of dementia, and given the cross-sectional studies showing associations and correlations of brain volume and cognitive dysfunction.^12,21^ The lacking association of MRI measures and SDMT change may be due to the properties of the SDMT as a longitudinal outcome measure. In a recent investigation in the ASCEND dataset, we found that, unexpectedly and in contrast to the physical outcome measures EDSS, T25FW and NHPT, SDMT performance steadily improved over course of the trial, possibly due to a practice effect.^22^ The SDMT may therefore not adequately reflect the steady cognitive decline that people with SPMS experience.

NBV loss was the only volume measure associated with a clinical outcome. NBV loss at 48 weeks was associated with NHPT worsening at 48 and 96 weeks, which may suggest that early NBV loss may have a protracted effect until 96 weeks. However, this association was only significant for NBV loss of more than 1%, which occurred in only 16.5% of trial participants; in itself, the relatively low percentage with this much loss in 96 weeks may not be surprising. However, this association was also inconsistent, since NBV loss at 48 weeks was associated with NHPT worsening, while NBV loss at 96 weeks was not; if NBV loss were an accurate reflection of chronic neurodegeneration, one would expect this association to remain or even to get stronger over time. In contrast to smaller longitudinal studies which found gray matter atrophy to be more prominent and more closely related to disability,^16–18^ we found no association of NCGMV or NWGMV with any clinical outcome. We showed previously that the NHPT is one of the more reliable clinical outcomes,^23^ but it is also the slowest to change among physical disability measures in SPMS.^24^ In this context, it is important to note that we found no relation between brain volume measures and T25FW performance, even though the T25FW is the most sensitive and possibly most useful clinical outcome in SPMS.^23,24^

The cNT2 at 96 weeks was associated with significant worsening of the T25FW at 48 and 96 weeks, and of the NHPT at 96 weeks. This association was significant for patients with more than ten cNT2, a group including only 9.6% of trial participants. Contrast enhancing lesions were not associated with any clinical outcome in this study. This is in keeping with the idea that disability worsening in SPMS is driven by different pathophysiological processes than relapsing-remitting MS,^25^ and largely independent of focal inflammatory demyelination.

Our findings suggest that while brain volume loss occurs and can be measured with MRI in patients with SPMS, the typical trial duration of 2 or 3 years is likely not sufficient for brain volume loss to manifest clinically. This raises the question whether phase 2 trials in SPMS, which aim at discovering new treatments to slow down or prevent irreversible worsening of disability, should rely on brain volume measures as their primary outcome.

There are several limitations to this study. First, we assess the changes in outcomes for only 96 weeks, a relatively short time period for both clinical and MRI outcomes. In our investigation we used only those measurements that are present at each time point, so that the “selective drop-out” of participants with especially severe MRI changes could have biased the results toward the null hypothesis. We examined this by comparing MRI outcomes at 48 weeks between individuals with and without a subsequent measurement at 96 weeks and no significant differences, which argues against a strong influence of “selective drop-outs” in this cohort.

We also need to keep in mind that MRI changes may precede their clinical manifestation. The study duration of 96 weeks could well be too brief to address this, however, within the confines of the trial duration, we did not see an association of MRI changes at 48 weeks with clinical outcome at 96 weeks, with the single exception of NBV change at 48 weeks predicting NHPT worsening at 96 weeks (Tables 4 and 5). The possible longer term effects of MRI outcomes on disability worsening should be investigated in other clinical trial datasets in progressive and relapsing-remitting MS.

The failure of MRI metrics to adequately predict clinical outcomes in SPMS may be due to the MRI not measuring important contributors to disability. For example, brain MRI does not evaluate spinal cord pathology. Similarly, although the investigated volume measures in this study are currently the most commonly used in clinical trials, newer MRI metrics such as thalamic^26^ or corpus callosum^27^ atrophy may have a closer relation to clinical outcome. Such newer MRI metrics should be investigated in other datasets. Axon and neuron death, which are believed to be important contributors to progression in MS, are a small component of brain volume and may be difficult to measure separately from other CNS components. The measurement of whole and gray matter volumes also depends on the technical details of the automatic segmentation and calculation methods used. SIENAX,^9^ the segmentation technique used in this study, is a well established and widely used method, however, it should be noted that the few studies comparing different segmentation techniques show meaningful differences in volume estimates between them,^28–30^ which makes the interpretation of brain atrophy measurements even more challenging.

The power of this study must be kept in mind. While the sample size of ASCEND is almost 900 participants, the number of participants with substantial changes on their MRIs is relatively small (Table 2). Showing mean changes in brain volume may not be a sufficient signal by which to judge a phase 2 trial since we do not know the time horizon to show clinical changes. ASCEND also had a relatively large number of participants, 26% of the cohort, drop out of the trial by the end of follow-up,^7^ which may have affected the precision of our analyses.

In sum, our investigation showed a disconnect between clinical outcomes and MRI measures in a large and well-characterized trial cohort in SPMS. Our findings call the current practice of using MRI changes as primary outcome measures in progressive MS trials into question. The association of clinical measures of disability worsening and MRI outcomes, and the possible predictive value of MRI changes beyond 96 weeks should be investigated in other trial datasets and clinical cohorts in progressive and relapsing-remitting MS.

## References

[1] Reich DS Lucchinetti CF Calabresi PA. Multiple sclerosis. N Engl J Med 2018; 378(2): 169–180. 29320652 10.1056/NEJMra1401483 PMC6942519

[2] Azevedo CJ Overton E Khadka S , et al. Early CNS neurodegeneration in radiologically isolated syndrome. Neurol Neuroimmunol Neuroinflamm 2015; 2(3): e102. 25884012 10.1212/NXI.0000000000000102 PMC4396526

[3] Lassmann H. Pathogenic mechanisms associated with different clinical courses of multiple sclerosis. Front Immunol 2018; 9: 3116. 30687321 10.3389/fimmu.2018.03116 PMC6335289

[4] Kapoor R Furby J Hayton T , et al. Lamotrigine for neuroprotection in secondary progressive multiple sclerosis: A randomised, double-blind, placebo-controlled, parallel-group trial. Lancet Neurol 2010; 9(7): 681–688. 20621711 10.1016/S1474-4422(10)70131-9

[5] Chataway J Schuerer N Alsanousi A , et al. Effect of high-dose simvastatin on brain atrophy and disability in secondary progressive multiple sclerosis (MS-STAT): A randomised, placebo-controlled, phase 2 trial. Lancet 2014; 383(9936): 2213–2221. 24655729 10.1016/S0140-6736(13)62242-4

[6] Chataway J De Angelis F Connick P , et al. Efficacy of three neuroprotective drugs in secondary progressive multiple sclerosis (MS-SMART): A phase 2b, multiarm, double-blind, randomised placebo-controlled trial. Lancet Neurol 2020; 19(3): 214–225. 31981516 10.1016/S1474-4422(19)30485-5 PMC7029307

[7] Kapoor R Ho P-R Campbell N , et al. Effect of natalizumab on disease progression in secondary progressive multiple sclerosis (ASCEND): A phase 3, randomised, double-blind, placebo-controlled trial with an open-label extension. Lancet Neurol 2018; 17(5): 405–415. 29545067 10.1016/S1474-4422(18)30069-3

[8] Roxburgh RHSR Seaman SR Masterman T , et al. Multiple sclerosis severity score: Using disability and disease duration to rate disease severity. Neurology 2005; 64(7): 1144–1151. 15824338 10.1212/01.WNL.0000156155.19270.F8

[9] Smith SM Zhang Y Jenkinson M , et al. Accurate, robust, and automated longitudinal and cross-sectional brain change analysis. NeuroImage 2002; 17(1): 479–489. 12482100 10.1006/nimg.2002.1040

[10] Morrow SA Drake A Zivadinov R , et al. Predicting loss of employment over three years in multiple sclerosis: Clinically meaningful cognitive decline. Clin Neuropsychol 2010; 24(7): 1131–1145. 20830649 10.1080/13854046.2010.511272

[11] R Core Team. R: A language and environment for statistical computing. Vienna: R Foundation for Statistical Computing, 2021, http://www.R-project.org/

[12] Sanfilipo MP Benedict RHB Weinstock-Guttman B , et al. Gray and white matter brain atrophy and neuropsychological impairment in multiple sclerosis. Neurology 2006; 66(5): 685–692. 16534104 10.1212/01.wnl.0000201238.93586.d9

[13] Sastre-Garriga J Ingle GT Chard DT , et al. Grey and white matter volume changes in early primary progressive multiple sclerosis: A longitudinal study. Brain 2005; 128(Pt 6): 1454–1460. 15817511 10.1093/brain/awh498

[14] University of California, San Francisco MS-EPIC Team, Cree BAC Hollenbach JA , et al. Silent progression in disease activity-free relapsing multiple sclerosis. Ann Neurol 2019; 85(5): 653–666. 30851128 10.1002/ana.25463 PMC6518998

[15] Eshaghi A Prados F Brownlee WJ , et al. Deep gray matter volume loss drives disability worsening in multiple sclerosis. Ann Neurol 2018; 83(2): 210–222. 29331092 10.1002/ana.25145 PMC5838522

[16] Fisniku LK Chard DT Jackson JS , et al. Gray matter atrophy is related to long-term disability in multiple sclerosis. Ann Neurol 2008; 64(3): 247–254. 18570297 10.1002/ana.21423

[17] Filippi M Preziosa P Copetti M , et al. Gray matter damage predicts the accumulation of disability 13 years later in MS. Neurology 2013; 81(20): 1759–1767. 24122185 10.1212/01.wnl.0000435551.90824.d0

[18] Jacobsen C Hagemeier J Myhr K-M , et al. Brain atrophy and disability progression in multiple sclerosis patients: A 10-year follow-up study. J Neurol Neurosurg Psychiatry 2014; 85(10): 1109–1115. 24554101 10.1136/jnnp-2013-306906

[19] Benedict RHB Carone DA Bakshi R. Correlating brain atrophy with cognitive dysfunction, mood disturbances, and personality disorder in multiple sclerosis. J Neuroimaging 2004; 14(Suppl. 3): 36S–45S. 15228758 10.1177/1051228404266267

[20] Kalb R Beier M Benedict RH , et al. Recommendations for cognitive screening and management in multiple sclerosis care. Mult Scler 2018; 24(13): 1665–1680. 30303036 10.1177/1352458518803785 PMC6238181

[21] Calabrese M Agosta F Rinaldi F , et al. Cortical lesions and atrophy associated with cognitive impairment in relapsing-remitting multiple sclerosis. Arch Neurol 2009; 66(9): 1144–1150. 19752305 10.1001/archneurol.2009.174

[22] Koch MW Mostert J Repovic P , et al. Is the Symbol Digit Modalities Test a useful outcome in secondary progressive multiple sclerosis? Eur J Neurol 2021; 28(6): 2115–2120. 33448539 10.1111/ene.14732

[23] Koch MW Mostert J Repovic P , et al. Reliability of outcome measures in clinical trials in secondary progressive multiple sclerosis. Neurology 2021; 96(1): e111–e120. 33106389 10.1212/WNL.0000000000011123

[24] Koch MW Mostert J Uitdehaag B , et al. Clinical outcome measures in SPMS trials: An analysis of the IMPACT and ASCEND original trial data sets. Mult Scler 2020; 26(12): 1540–1549. 31517591 10.1177/1352458519876701

[25] Mahad DH Trapp BD Lassmann H. Pathological mechanisms in progressive multiple sclerosis. Lancet Neurol 2015; 14(2): 183–193. 25772897 10.1016/S1474-4422(14)70256-X

[26] Azevedo CJ Cen SY Khadka S , et al. Thalamic atrophy in multiple sclerosis: A magnetic resonance imaging marker of neurodegeneration throughout disease. Ann Neurol 2018; 83(2): 223–234. 29328531 10.1002/ana.25150 PMC6317847

[27] Papathanasiou A Messinis L Zampakis P , et al. Corpus callosum atrophy as a marker of clinically meaningful cognitive decline in secondary progressive multiple sclerosis. Impact on employment status. J Clin Neurosci 2017; 43: 170–175. 28601572 10.1016/j.jocn.2017.05.032

[28] Steenwijk MD Amiri H Schoonheim MM , et al. Agreement of MSmetrix with established methods for measuring cross-sectional and longitudinal brain atrophy. Neuroimage Clin 2017; 15: 843–853. 28794970 10.1016/j.nicl.2017.06.034 PMC5540882

[29] Derakhshan M Caramanos Z Giacomini PS , et al. Evaluation of automated techniques for the quantification of grey matter atrophy in patients with multiple sclerosis. NeuroImage 2010; 52(4): 1261–1267. 20483380 10.1016/j.neuroimage.2010.05.029

[30] Eggert LD Sommer J Jansen A , et al. Accuracy and reliability of automated gray matter segmentation pathways on real and simulated structural magnetic resonance images of the human brain. PLoS ONE 2012; 7(9): e45081. 23028771 10.1371/journal.pone.0045081 PMC3445568

